# Antimicrobial, antibiofilm, and microbial barrier properties of poly (ε-caprolactone)/cloisite 30B thin films

**DOI:** 10.1007/s13205-016-0559-7

**Published:** 2016-11-18

**Authors:** Snigdha Sajeendra Babu, Shiji Mathew, Nandakumar Kalarikkal, Sabu Thomas, Radhakrishnan E. K

**Affiliations:** 1 International and Inter University Centre for Nanoscience and Nanotechnology, Mahatma Gandhi University, Kottayam, 686 560 India; 2 School of Biosciences, Mahatma Gandhi University, Kottayam, 686 560 India; 3 School of Pure and Applied Physics, Mahatma Gandhi University, Kottayam, 686 560 India; 4 School of Chemical Sciences, Mahatma Gandhi University, Kottayam, 686 560 India

**Keywords:** Microbial barrier, Antimicrobial, Antibiofilm, PCL/cloisite 30B, SEM, AFM, Mechanism of antimicrobial activity, Packaging, Nanoclay, AFM of PCL/Clay

## Abstract

Development of antibacterial and antibiofilm surfaces is in high demand. In this study, nanocomposite of Poly (ε-caprolactone)/Cloisite 30B was prepared by the solvent casting method. The membranes were characterised by SEM, AFM, and FTIR. Evaluation of water uptake, antimicrobial, antibiofilm, and microbial barrier properties demonstrated a significant antimicrobial and antibiofilm activity against MTCC strain of *Staphylococcus haemolyticus* and strong biofilm positive *Staphylococcus epidermidis* of clinical origin at low clay concentrations. These membranes acted as an excellent barrier to the penetration of microorganism. These nanocomposites can have promising applications in various fields including packaging.

## Introduction

The development of antibiotic resistance in microbes (Neu 1992; Stewart and Costerton 2001) has turned into a major issue in the healthcare sector. As microorganisms are developing resistance to all the major antibiotics, there is an increased demand for the development of alternative methods for resisting microbial infections (Chatterjee et al. 2016). Nanostructured materials have gained a lot of popularity in biomedical applications due to their broad antibacterial, antimycotic, and antiparasitic activities. Major attractions with the use of nanomaterials include their size and surface area-based enhanced activity, easy penetration into the cells, and most remarkably their multi-targeted action. In addition, performance of various materials used for biomedical applications has significantly been improved by the addition of nanoparticles. Hence, exploration of antimicrobial properties of various nanomaterials is significantly important to generate cost-effective, biodegradable, and non-toxic anti-infective surfaces. As the very basic step that determines the survival and growth of microorganisms is its attachment to surface, prevention of this step can have significant medical and industrial applications.

With the advancement in nanotechnology, biodegradable polymers like poly(ε-caprolactone) have been exploited diversely for the development of various types of scaffolds, biological implants, drug delivery materials, sutures, and also for food packaging (Cabedo et al. 2006; Fukushima et al. 2010; Hsu 2016; Kweon et al. 2003; Lim et al. 2012; Qin et al. 2016; Venugopal and Ramakrishna 2005). PCL has also widely been used for the development of nanocomposites by incorporating various bioactive nanoparticles. The biological advantages of PCL include its biodegradability, stability under abiotic conditions and non-toxicity (Hakkarainen 2002; Pranamuda et al. 1996). In this study, cloisite 30B was selected as the candidate material to explore its performance in a thin film made of PCL matrix. Polymer nanocomposites based on clay/layered silicates have promising applications due to the ease of availability and preparation (Alexandre and Dubois 2000; Gorrasi et al. 2002).

Various organic moieties are introduced into clay to increase its compatibility with polymer and also for better dispersion. Cloisite 30B used in this study is an organoclay derived from montmorillonite by modification with methyl, tallow, bis-2-hydroxyethyl, quaternary ammonium, where tallow is ~65% C18; ~30% C16; ~5% C14. Although cloisite 30B has been shown to impart antimicrobial activity (de Azeredo 2013; Nigmatullin et al. 2009; Parolo et al. 2011) to polymeric materials, only limited reports are there on its antibiofilm activity. Therefore, the PCL incorporated Cloisite 30B has been studied for its antimicrobial and antibiofilm properties against Staphylococci (Babu et al. 2016). Specifically, the test organisms selected for the study were MTCC strain of *Staphylococcus hemolyticus* and clinical isolate of *Staphylococcus epidermidis.* As these organisms are notorious for the presence of an array of factors responsible for attachment to both biotic and abiotic surfaces, they form promising candidates to check the anti-infective properties of newly developed nanomaterials. The results of the study indicate the potential of synthesised PCL/Cloisite 30B thin-film membranes to have applications ranging from one-time use for biomedical package to a long-term package for commodity application. As PCL/Cloisite 30B thin film has not been well studied previously for these applications, the study is novel in its approach.

## Methodology

### Sample preparation

Solvent casting: 10 wt % solution of PCL (Mw 70,000, Sigma Aldrich, St. Luis, USA) in chloroform (Merck, Mumbai, India) was prepared and was mechanically stirred for 18 h to ensure uniform mixing and dissolution of polymer chains. Solutions with 1, 3, and 5% Cloisite 30B (C30B Clay, imported from Rockwood clay additives GmbH, Germany) in 10 wt % PCL, were prepared as mentioned above. These membranes were then poured into petriplates and swirled to ensure uniform distribution and it was closed with a lid to allow evaporation of solvent (Dottori et al. 2011). After the complete evaporation, the thin membranes obtained were labeled as PCC0, PCC1, PCC3, and PCC5 for PCL neat, PCL + 1% C30B, PCL + 3% C30B, and PCL + 5% C30B, respectively. These membranes were then dried in the hot air oven at 40 °C for 18 h and subjected to various characterizations.

### Characterization studies

#### Scanning electron microscopy (SEM)

The structural morphology of the electrospun membranes was studied using Scanning Electron Microscopy (SEM-JEOL 6390). The membranes were carefully sectioned into grids of dimension 3 × 0.5 mm and mounted on an SEM grid; the samples were coated with platinum prior to examination. SEM was carried out for neat PCL and PCL/Cloisite 30B composites.

#### Atomic force microscopy (AFM)

Surface topography of samples PCC0 and PCC5 in air was studied using atomic force microscope A-100 SPM, APE Research Nanotechnology, Italy. Atomic force microscopy (AFM) images were obtained by scanning surface in a contact mode with scan size 10 × 10 µm and scan rate 0.95–1.00 Hz. An arithmetic mean of the surface average roughness (Ra) was evaluated directly from the AFM images. The root-mean-square average of all the peaks (Rms) in a given area in the sample was also calculated.

#### Fourier transform infrared spectroscopy (FTIR)

Fourier transform infrared spectroscopy (FTIR, Vertex 80v, Bruker Optics) spectra of the samples were measured from 800 to 1600 cm^−1^ with a 4 cm^−1^ resolution and the co-addition of 64 scans were recorded.

#### Contact angle measurements (CA)

Contact angle (CA) measurements were carried out using Contact Analyser, Phoenix 300 from Surface Electro Optics Co. Ltd, Korea. CA of water in air was measured after a water drop was placed on the surface of the membrane using a micro syringe. All membranes were attached on the movable sample platform and levelled horizontally before measurements. The CA on both sides of the drop was measured to check symmetry and horizontal level. The contact angle was measured within 45–60 s of the addition of the liquid drop with an accuracy of ±2°. Measurements were repeated six to ten times with different test pieces of the same sample to check the accuracy. In addition, contact angles were measured for a single drop and the measurements were recorded as snap shots. The surface energy of the samples was obtained from the contact angle measurements from the instrument software.

#### Water uptake studies

The solvent cast membranes synthesised was cut into 4 mm diameter circles weighed and immersed in 5 mL distilled water at room temperature. These membranes were taken out of water, surface water was removed and weighed at intervals of 1, 3, 7, 14, and 30 days. The experiment was performed in triplicates. The amount of water absorbed was expressed as percentage water uptake and was calculated as follows:

1 $${\text{Water}}\;{\text{uptake}}\;(\% ) = \frac{{w_{t} - w_{0} }}{{w_{0} }} \times 100$$

where *W*_0_ is the initial weight of the solvent cast membrane and *W*_*t*_ is the weight of the solvent cast membrane at a given time *t* (Pérez et al. 2008).

#### Antibacterial activity of PCL/Cloisite 30B nanocomposites

The solvent cast membranes were evaluated for their antibacterial potential against selected strains by the disc diffusion method in Muller Hinton Agar (MHA) plates as per previous reports (Rhim et al. 2009). MTCC strain of *Staphylococcus hemolyticus* and a strong biofilm positive clinical isolate of *S. epidermidis* were selected as test organisms. Pure cultures of the test strains were grown in Trypticase Soy broth (TSB) at 37 °C for 18–24 h. Muller Hinton agar plates were prepared and these plates were inoculated by swabbing the test organism when its growth has reached to a turbidity equivalent to that of a 0.5 McFarland Standard. Then nanocomposite membranes were cut into discs of around 4 mm diameter, UV sterilised, and were placed on Muller Hinton agar plates using sterile forceps. The experiment was performed in triplicate and diameter of zone of inhibition in millimetre around each well was measured after incubation at 37 °C for 24 h.

#### Antibiofilm activity of PCL/Clay nanocomposites by Tissue culture plate method (TCP)—in vitro biofilm formation assay

To assess the ability of nanocomposite films to resist biofilm formation, strong biofilm positive clinical isolate of *S. epidermidis* was selected as the test organism. Bacteria was inoculated into TSB medium and incubated at 37 °C for 24 h under shaking. The cultures were serially diluted 1:100 in fresh TSB medium supplemented with 1% glucose. Then UV sterilised nanocomposite membrane discs of varying concentrations of Cloisite 30B (PCC0, PCC1, PCC3, and PCC5) were introduced into the corresponding wells of 96-welled polystyrene microtiter plates. Then, 200 µL each of the bacterial inoculum was transferred to the respective wells and the microtiter plate was incubated at 37 °C for 18 h. After incubation, the turbidity was recorded at 600 nm using microplate reader (Thermo scientific Varioskan Flash Multimode reader) and optical density (OD) was used as a measure of cell density in the culture media.

2 $$\frac{{N_{\text{Sample}} }}{{V_{\text{Sample}} }}\sim {\text{OD}}$$

where is the cell number per unit volume. From the OD values, the percentage of biofilm inhibition was calculated by the formula

3 $$\% \;{\text{of}}\;{\text{inhibition}} = \frac{{{\text{OD}}_{} {\text{control}} - {\text{OD}}_ {\text{treated}}}}{{{\text{OD}}_{} {\text{control}}}} \times 100.$$

Then, the media and the unattached cells were aspirated out, and the wells were washed 3–4 times with phosphate buffer saline (PBS). Then, the attached cells (biofilm) were fixed by heating at 60 °C for 60 min. The attached cells were then stained with 0.06% crystal violet stain for 5 min. Excess stain was rinsed off by washing with PBS 3–4 times and then the plates were kept for air drying. For the quantification of antibiofilm activity of nanocomposite discs, the adherent bacteria associated with crystal violet were solubilized with 95% ethanol and the absorbance was recorded at 600 nm using microplate reader (Thermo scientific Varioskan Flash Multimode reader). This OD value was considered as an index of bacterial ability to adhere to surface and form biofilms (Christensen et al. 1985). The % inhibition of biofilm was calculated using Eq. (3). This assay was performed in triplicate. The average value and the standard deviation of the data were calculated and were compared using the Tukey test. *p* value less than 0.05 was considered statistically significant.

#### Microbial barrier properties of PCL/Clay nanocomposites

The solvent cast membranes were cut into circles of 2.2 cm diameter and surface sterilised by treating with 70% ethanol followed by UV irradiation. Screw cap glass vials were autoclaved with 5 mL nutrient broth. The sterilised membranes were aseptically placed on the opening of the screw cap vial containing the nutrient broth and the edges were sealed with parafilm. Thus, the opening was covered with the nanocomposite membranes. The mouth of the control vial was left open. All the vials were incubated at room temperature on the lab shelf. The vials were observed after 7 days of incubation and 100 µL of the nutrient broth from each vial was plated onto sterilised petridishes containing nutrient agar medium with the help of L-shaped glass rod and incubated at room temperature for 24 h (Augustine et al. 2015).

## Results

### Scanning electron microscopy (SEM)

**Fig. 1:**
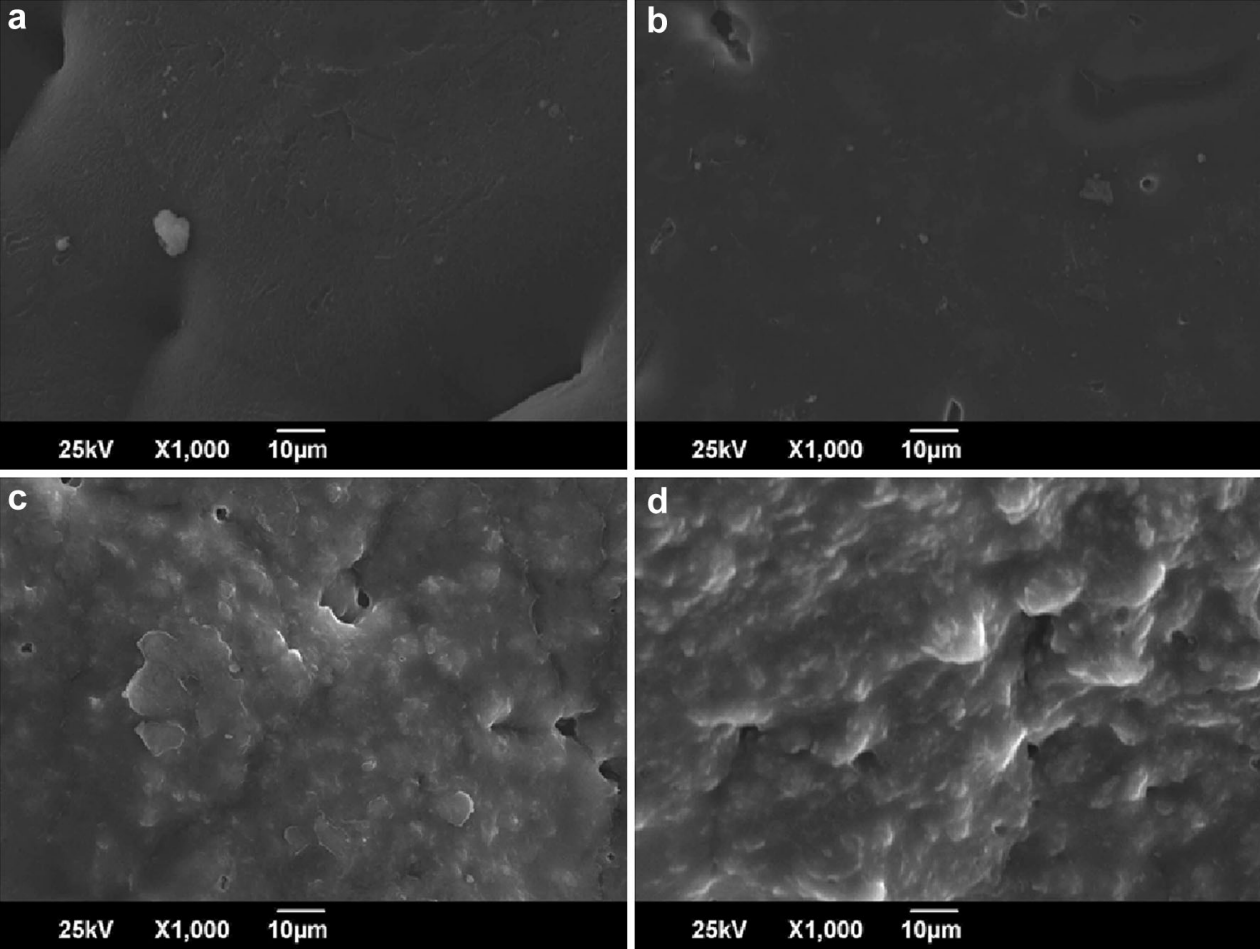
Scanning electron micrographs of **a** PCC0, **b** PCC1, **c** PCC3, and **d** PCC5

PCL films showed an uneven but smooth surface, with increase of filler concentration from PCC0 to PCC5, the surface morphology transitions from smooth to rugged with the PCC5 having highly uneven and rugged surface. The light colored clumps on the membrane surfaces of PCC3 and PCC5 showed the presence of clay particles (Fig. 1).

### Atomic force microscopy (AFM)

**Fig. 2:**
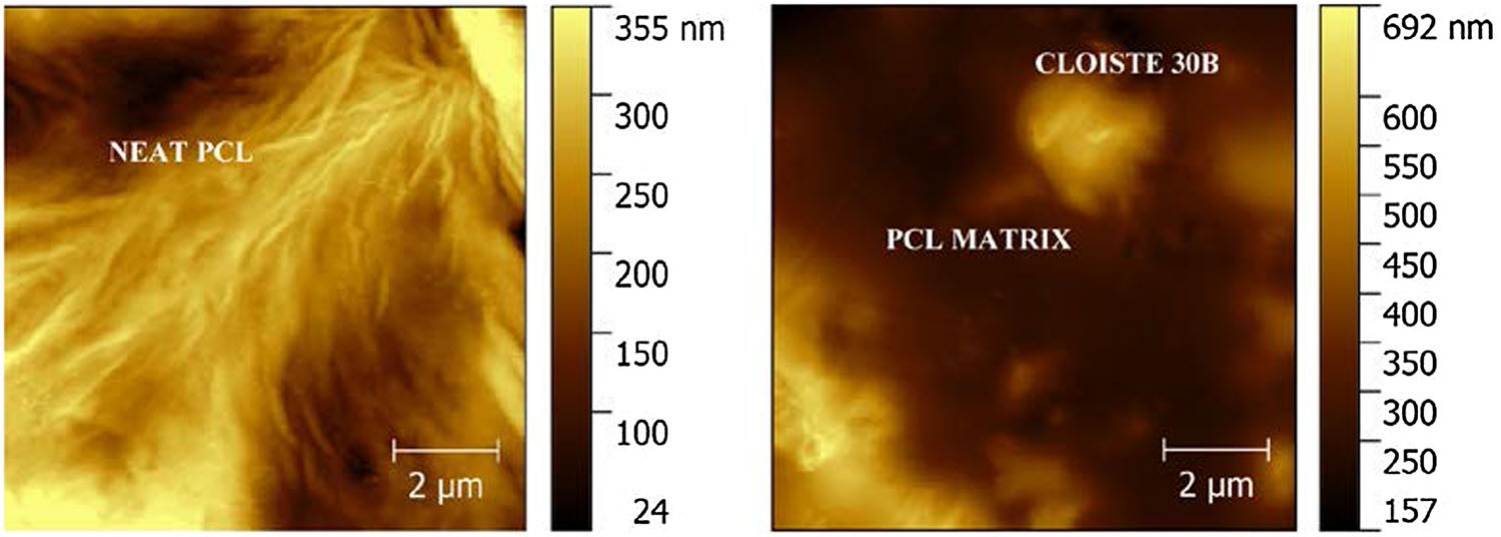
Topographic AFM images of **a** PCC0 and **b** PCC5

The surface topography of membranes was studied using AFM (Fig. 2). Dense uniaxially oriented fibrils of PCL were observed in PCC0, which are characteristic crystallite structures of PCL (Cheng and Teoh 2004; Lim et al. 2012; Ng et al. 2000). The Ra and Rms values were calculated from the roughness profile determined by AFM. The Ra of unmodified PCL film surface was 51.59 nm.

### Fourier transform infrared spectroscopy (FTIR)

**Fig. 3:**
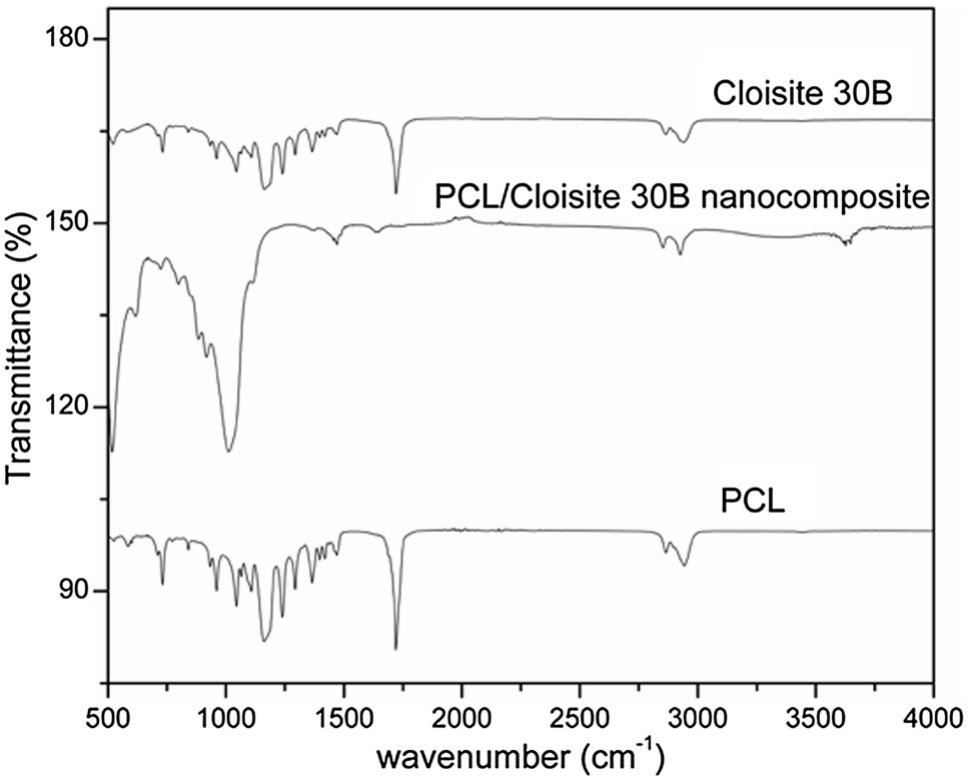
FTIR spectra of PCC0, Cloisite 30B, and nanocomposite (NC)

The FTIR spectra of PCL, cloisite 30B, and PCL/C30B nanocomposite were analysed (Fig. 3). The peaks for PCL were at 2943, 2865, and 1720 cm^−1^. This represents the stretching vibration of –CH_2_ and vibration of –C=O bonds, respectively, these peaks showed a decrease in intensity in the PCL/Cloisite 30B nanocomposite. For Cloisite 30b, Si–AO bending peaks were identified at 520 and 467 cm^−1^, but these peaks have been masked in the PCL/Cloisite 30B nanocomposite. The Si–AO stretching peaks could be seen at 1086 and 1034.

### Water uptake studies

**Fig. 4:**
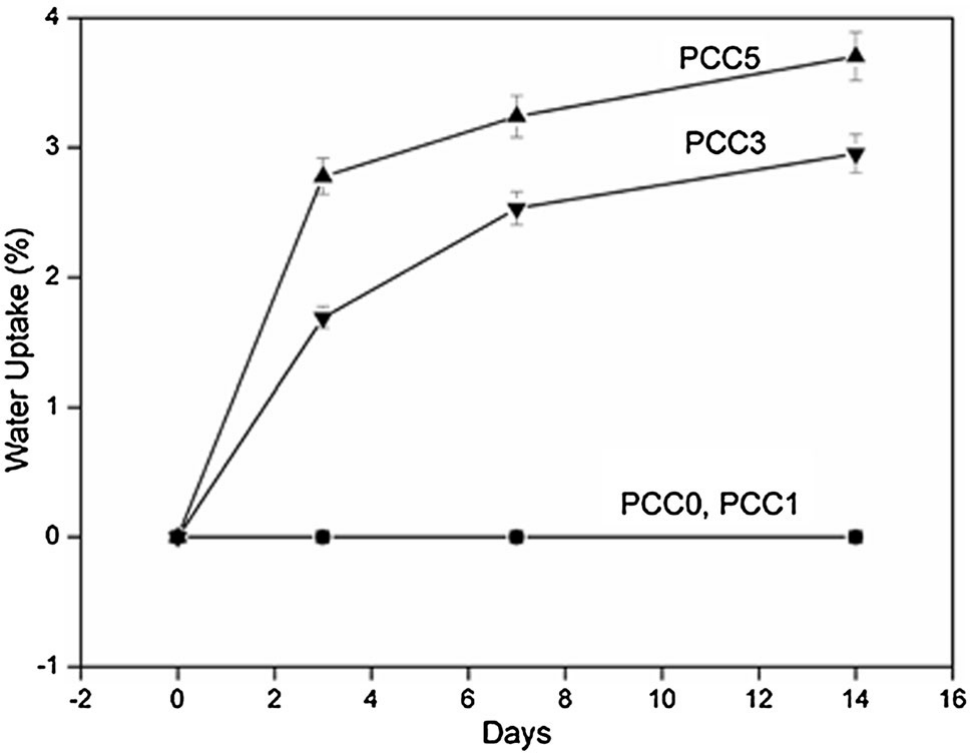
Water uptake plots of PCC0, PCC1, PCC3, and PCC5

Water uptake studies showed that PCC0 and PCC1 did not show any water uptake as their weights remained constant throughout the period of study. Thus, PCC0 and PCC1 do not absorb water. The PCC3 and PCC5 showed consistent increase in water absorption up to day 3 and then the water uptake tends to slow down and remained stationary at around 2 and 4%, respectively. It can be observed that the membranes do not show any significant change in water uptake capacity upon increasing filler addition (Fig. 4). The experiment was performed in triplicate.

### Contact angle measurements (CA)

**Fig. 5:**
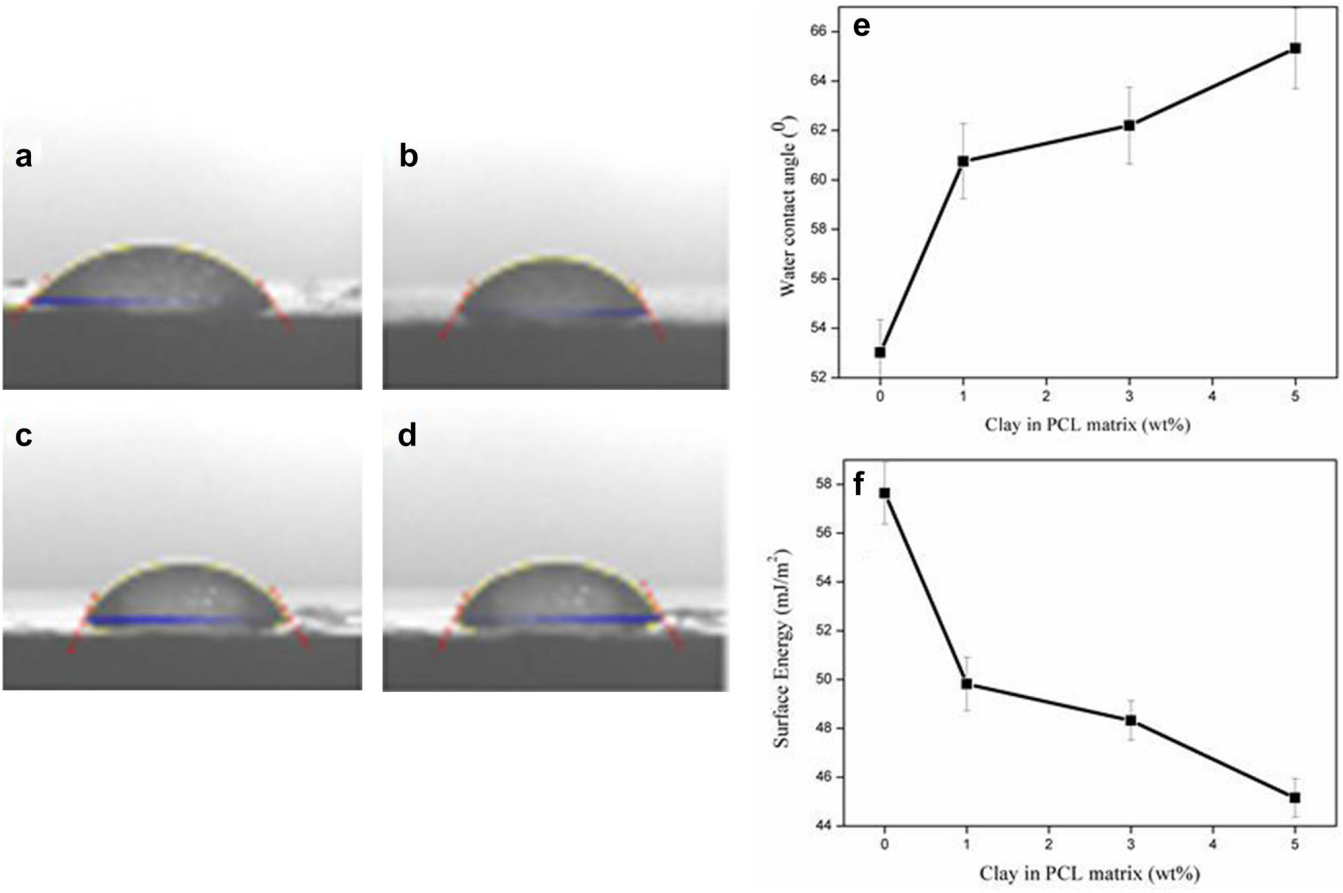
Water contact angle (CA) images of **a** PCC0, **b** PCC1, **c** PCC3, **d** PCC5, **e** clay loading versus surface energy, and **f** clay loading versus contact angle

The contact angle was found to increase with the increase of filler loading (Fig. 5a–e). There was a tenfold increase in contact angle with the addition of 5% filler. The surface energy of the nanocomposite film has been found to be decreasing with increase in clay concentration (Fig. 5f, Table 1).

**Table 1:** Contact angle studies of PCL/Clay composite

Sample	Contact angle (°) ±2°	Work of adhesion	Spreading coefficient	Surface energy
PCC0	53.02	116.5851	29.01486	57.63
PCC1	60.75	107.5021	38.09787	49.82
PCC3	62.20	106.748	38.85197	48.32
PCC5	65.32	103.0467	42.55332	45.15

### Antibacterial activity of PCL/clay nanocomposites

**Fig. 6:**
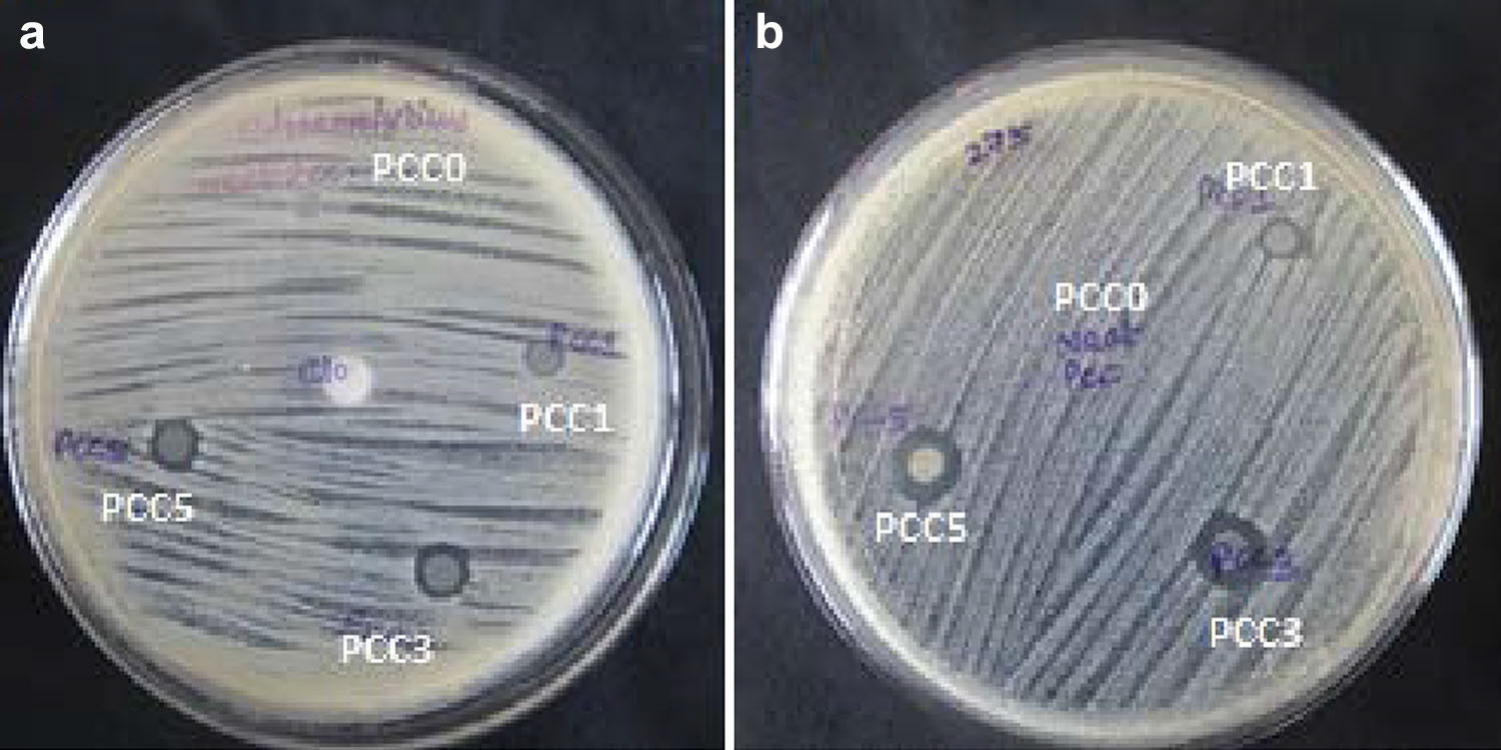
Microbicidal activity of the composites against **a** *S. haemolyticus*, **b** *S. epidermidis*

The synthesised membranes showed a filler concentration-dependent antibacterial activity against *Staphylococcus haemolyticus* and *S. epidermidis* (Fig. 6). No antibacterial activity was seen with PCC0 against any of the tested organisms. The zone of inhibition against *S. epidermidis* was found to be 6 mm for PCC1, 10 mm for PCC3, and 13 mm for PCC5. For *S. haemolyticus*, the same was 4 mm in the case of PCC1, 9 mm for PCC3 and 10 mm for PCC5.

### Antibiofilm activity of PCL/Clay nanocomposites by tissue culture plate method (TCP)

Biofilm detachment studies of nanocomposite films were carried out in vitro in 96-well plate. The results were quantified and a significant decrease in turbidity was observed. Upon measuring OD at 600 nm, the unstained wells showed 61, 83, and 89.5% inhibition in PCC1, PCC3, and PCC5, respectively. In the wells stained with crystal violet, no significant inhibition was identified in the biofilm formation with PCC0 and PCC1. However, PCC3 and PCC5 showed percent inhibition of 16 and 22%, respectively.

### Microbial barrier properties of PCL/Clay nanocomposites

**Fig. 7:**
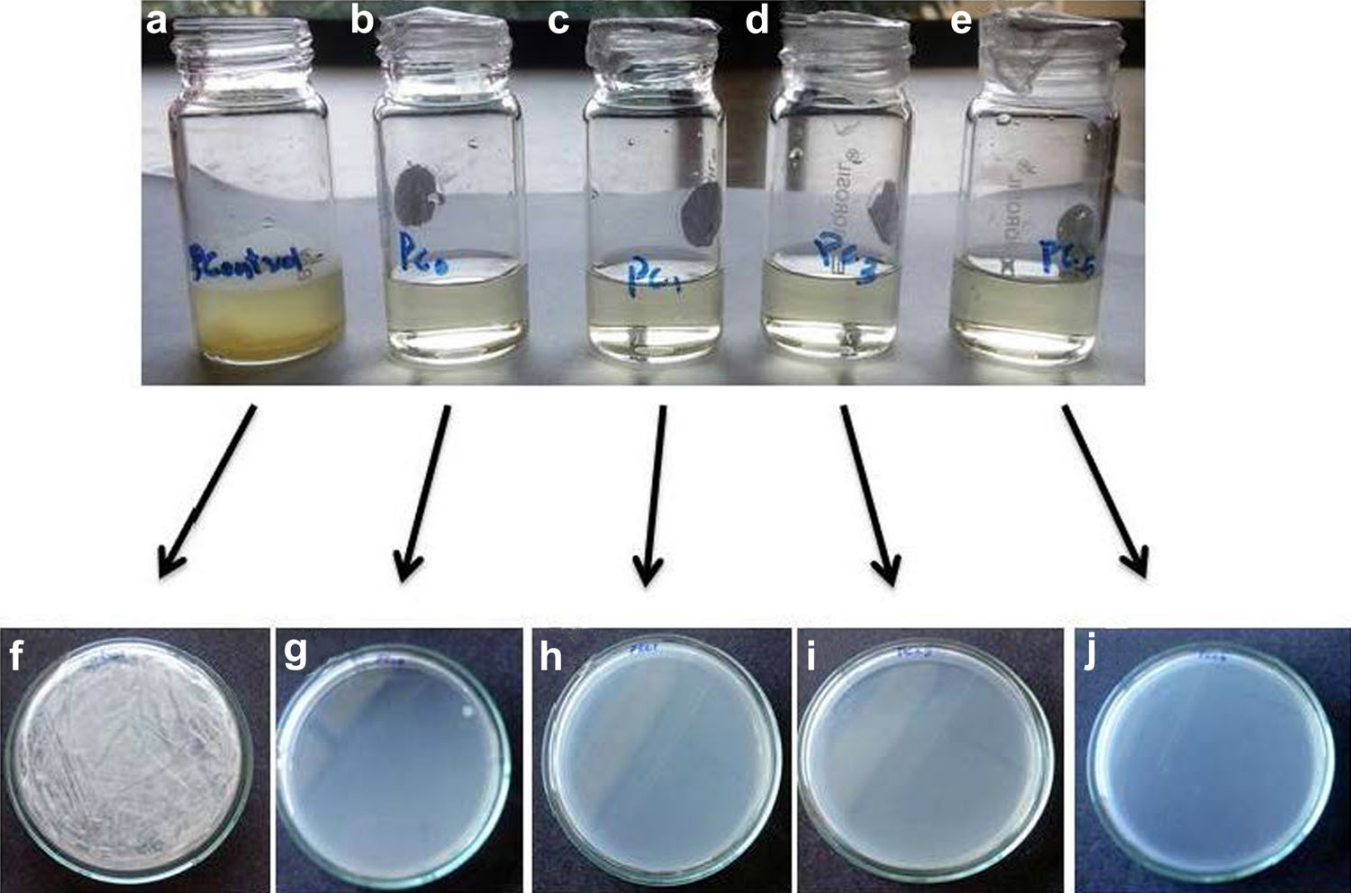
Microbial barrier property of **a** uncovered media, media covered with **b** PCC0, **c** PCC1, **d** PCC3, **e** PCC5, spread plate of **f** uncovered media, **g**PCC0, **h** PCC1, **i** PCC3, and **j** PCC5

Microbial barrier properties of the PCL/Clay nanocomposites were carried out to study the level of penetration of environmental microorganisms through the membranes. From Fig. 7, it can be observed that the screw cap vials that were covered with PCC0, PCC1, PCC3, and PCC5 do not show any visible change in turbidity, whereas the control vial had developed very significant amount of turbidity.

The media from the various vials were spread on sterile nutrient agar medium and incubated for 24 h. The plate with the inoculum from the control vials showed dense growth of microorganisms; from PCC0 covered vial, two isolated microbial colonies were observed and the other membranes showed no microbial growth.

## Discussion

In this study, PCL/cloisite 30B composites were prepared and their properties were studied. From the SEM images, the surface of membranes can be observed to turn from smooth to rugged with increase in filler concentration. From the AFM images, nanoscale roughness of the membranes surfaces was measured as Ra and dense uniaxially oriented fibrils of PCL were observed in PCC0 as its characteristic feature (Cheng and Teoh 2004; Ng et al. 2000). The fibril formation has decreased in PCC5, which could indicate possible prevention of PCL nucleation by clay platelets. The Ra value was calculated from the roughness profile determined by AFM. The Ra of unmodified PCL film surface was 51.59 nm. After filler addition, the Ra increased to 69.54 nm for PCC5. The Rms values calculated from the AFM images were 67.56 nm and 96.58 nm for PCC0 and PCC5, respectively. There is a significant increase in Rms values after filler addition. The increased Ra and Rms values correspond to the morphological change observed in the SEM images (Diaconu et al. 2008). From the FTIR spectra, the incorporation of Cloisite 30B in the PCL matrix could be confirmed (Yahiaoui et al. 2015). From the results of water uptake studies, it can be observed that the membranes do not show any significant change upon increasing filler addition. Hence, this membrane can perform well in aqueous environment without any change in membrane integrity.

The contact angle measurements showed an increase in value as a function of filler loading. The hydrophobic filler which was added to the system might have increased the contact angle and thereby the hydrophobicity of the composite film. There was a tenfold increase in contact angle with the addition of 5% filler. From the mechanistic point of view, the hydrophobicity of the synthesised membrane could also aid in the antimicrobial activity by attracting the microbes to the surface of the composites. The previous studies have reported *S. aureus* to have hydrophobic characteristics (contact angle *θ* = 72 °C) due to the presence of highly negatively charged teichoic acid and lipoteichoic acid in its cell wall (Mitik-Dineva et al. 2009). Thermodynamically, hydrophobic cells would show preference for hydrophobic surfaces (Bos et al. 1999; Bruinsma et al. 2001). Hence, the bacteria might get attracted towards the hydrophobic membranes and subsequently get killed by the free surfactant present on the surface of the membrane. The surface energy of the composite film has been found to be decreasing with increase in clay concentration (Fig. 5f, Table 1); this indicates that the system developed the tendency to become less reactive with the surrounding when compared to the thin film without any filler. Upon lowering the free energy of the system, the polymer chains preferentially interact with the filler surface, and thereby decreasing the interaction with the surroundings. As with the contact angle, the surface energy also showed a tenfold increase in value of PCC5 (Abraham et al. 2012).

From the results of the study, the solvent cast membranes were found to exhibit filler concentration-dependent inhibition activity against *S. haemolysis* and *S. epidermidis*. As the antimicrobial activity is absent in PCC0, activity observed in PCC1, PCC3, and PCC5 can be confirmed to be due to the presence of cloisite 30B (Speranza et al. 2004). The antimicrobial activity of the PCL/clay composite has been linked to the migration of free ammonium surfactant from PCL composite film to the culture media, because intercalation of the PCL into the clay galleries could cause the release of the ammonium surfactant associated with the negatively charged part of the clay (Ferfera‐Harrar et al. 2014; Malachová et al. 2009). Hence, microbicidal activity of the composite can be considered to be related to the ammonium surfactants of the organoclay (Eudy 1981; Sauvet et al. 2000; Senuma et al. 1989) which can have activity against microorganisms due to their ability to interact with cell membrane (Yahiaoui et al. 2015). Under physiological conditions, bacterial cell wall has a negative charge due to the presence of functional groups, such as carboxylates, present in lipoproteins at the surface (Breen et al. 1995). The charge on clay minerals and the alkyl ammonium surfactant tends to attract the bacteria through electrostatic interactions. Hydrophobic interactions may also be involved between hydrophobic components of Cloisite 30B and the lipophilic components of bacterial cell walls, such as lipoproteins, liposaccharides, and phospholipids. Inactivation of bacteria may involve killing them or making them incapable of replicating (Herrera et al. 2000). It was suggested that both the charge and hydrophobic nature of the organoclay favour the association of bacteria to the surface of organoclay, where the surfactant performs its antimicrobial activity.

The biofilm detachment in the presence of composite films was carried out in vitro in 96-well plate. The significant reduction in the observed bacterial biomass can be an indication of ability of Cloisite 30B to kill the microbes and interfere with microbial replication. The results were quantified and no significant change in the biofilm formation was observed in PCC0 and PCC1. However, PCC3 and PCC5 showed a percent inhibition of biofilm formation to 16 and 22%, respectively. The decrease in cell attachment can be explained in terms of inability of microbial cells to replicate and due to cell death. (Herrera et al. 2000).

The composite membranes containing Cloisite 30B as filler proved to be very efficient barriers against microorganism when compared to uncovered media and PCC0 (Fig. 7). This property can be attributed to the fact that PCL/clay composites prevented the penetration of environmental microflora into the nutrient media. The filler in the composite, which has already been shown as a good antimicrobial candidate, could have asserted its antimicrobial property before the microbes could penetrate the membrane and grow in the contained medium. Thus, this membrane could be a very effective packaging material against microbial fouling of the packaged material, since even very low concentrations of the nanoclay prevented microbial penetration.

## Conclusion

The PCL/Clay composite films are promising candidates for biomedical and packaging applications due to its antibiofilm, anti-infective nature, and excellent microbial barrier properties. In this study, the thin films with organoclays effectively prevented the formation of microbial biofilms on their surface, exhibited antibacterial activity, and acted as excellent barrier against the penetration of microorganism from the environment. There was a significant decrease in microbial biomass and biofilm formation even at low concentration of organoclay (5 wt %). The remarkable barrier property of the composite at very low filler concentration (1 wt %) is also notable. Therefore, this composite system could be further explored for its biomedical and industrial applications where the biofilm formation and microbial fouling are of great concern.

## Abbreviations

SEM: Scanning electron microscopy
AFM: Atomic force microscopy
FTIR: Fourier transform infrared spectroscopy
MTCC: Microbial type culture collection

## References

[CR1] Abraham R, Varughese KT, Isac J, Thomas S (2012). Wetting properties of barium sodium niobate filled polystyrene nanocomposite. Macromol Symp.

[CR2] Alexandre M, Dubois P (2000). Polymer-layered silicate nanocomposites: preparation, properties and uses of a new class of materials. Mater Sci Eng R Rep.

[CR3] Augustine R, Kalarikkal N, Thomas S (2015). An in vitro method for the determination of microbial barrier property (MBP) of porous polymeric membranes for skin substitute and wound dressing applications. Tissue Eng Regen Med.

[CR4] Babu SS, Augustine A, Kalarikkal N, Thomas S (2016). Nylon 6, 12/Cloisite 30B electrospun nanocomposites for dental applications. J Sib Fed Univ Biol.

[CR5] Bos R, Van der Mei HC, Busscher HJ (1999). Physico-chemistry of initial microbial adhesive interactions—its mechanisms and methods for study. FEMS Microbiol Rev.

[CR6] Breen PJ, Compadre CM, Fifer E, Salari H, Serbus DC, Lattin DL (1995). Quaternary ammonium compounds inhibit and reduce the attachment of viable Salmonella typhimurium to poultry tissues. J Food Sci.

[CR7] Bruinsma GM, Rustema-Abbing M, van der Mei HC, Busscher HJ (2001). Effects of cell surface damage on surface properties and adhesion of Pseudomonas aeruginosa. J Microbiol Methods.

[CR8] Cabedo L, Luis Feijoo J, Pilar Villanueva M, Lagarón JM, Giménez E (2006). Optimization of biodegradable nanocomposites based on aPLA/PCL blends for food packaging applications. Macromol Symp.

[CR9] Chatterjee M, Anju C, Biswas L, Kumar VA, Mohan CG, Biswas R (2016). Antibiotic resistance in *Pseudomonas aeruginosa* and alternative therapeutic options. Int J Med Microbiol.

[CR10] Cheng Z, Teoh S-H (2004). Surface modification of ultra thin poly (ε-caprolactone) films using acrylic acid and collagen. Biomaterials.

[CR11] Christensen GD, Simpson W, Younger J, Baddour L, Barrett F, Melton D, Beachey E (1985). Adherence of coagulase-negative staphylococci to plastic tissue culture plates: a quantitative model for the adherence of staphylococci to medical devices. J Clin Microbiol.

[CR12] de Azeredo H (2013). Antimicrobial nanostructures in food packaging. Trends Food Sci Technol.

[CR13] Diaconu G, Paulis M, Leiza JR (2008). High solids content waterborne acrylic/montmorillonite nanocomposites by miniemulsion polymerization. Macromol React Eng.

[CR14] Dottori M, Armentano I, Fortunati E, Kenny J (2011). Production and properties of solvent-cast poly (ε-caprolactone) composites with carbon nanostructures. J Appl Polym Sci.

[CR15] Eudy WW (1981) Organosilicon quaternary ammonium antimicrobial compounds. Google Patents

[CR16] Ferfera-Harrar H, Aiouaz N, Dairi N, Hadj-Hamou AS (2014) Preparation of chitosan-g-poly(acrylamide)/montmorillonite superabsorbent polymer composites: studies on swelling, thermal, and antibacterial properties. J Appl Polym Sci 131(1). doi:10.1002/app.39747

[CR17] Fukushima K, Abbate C, Tabuani D, Gennari M, Rizzarelli P, Camino G (2010). Biodegradation trend of poly (ε-caprolactone) and nanocomposites. Mater Sci Eng C.

[CR18] Gorrasi G, Tortora M, Vittoria V, Galli G, Chiellini E (2002). Transport and mechanical properties of blends of poly (ϵ-caprolactone) and a modified montmorillonite-poly (ϵ-caprolactone) nanocomposite. J Polym Sci Part B Polym Phys.

[CR19] Hakkarainen M (2002) Aliphatic polyesters: abiotic and biotic degradation and degradation products. In: Albertsson AC (ed) Degradable aliphatic polyesters. Springer, Berlin, Heidelberg, pp 113–138

[CR20] Herrera P, Burghardt R, Phillips T (2000). Adsorption of Salmonella enteritidis by cetylpyridinium-exchanged montmorillonite clays. Vet Microbiol.

[CR21] Hsu CY, Hsiao JP, Shieh MJ, Lai PS (2011) Nanotech 2011: siRNA and Paclitaxel Loaded PEG-PCL-PEI Tri-block Polymeric Micelle for Gene and Chemo-therapy Applications in Multidrug-resistence cancer cells, vol 3, pp 225–228

[CR22] Kweon H (2003). A novel degradable polycaprolactone networks for tissue engineering. Biomaterials.

[CR23] Lim JS, Ki CS, Kim JW, Lee KG, Kang SW, Kweon HY, Park YH (2012). Fabrication and evaluation of poly (epsilon-caprolactone)/silk fibroin blend nanofibrous scaffold. Biopolymers.

[CR24] Malachová K, Praus P, Pavlíčková Z, Turicová M (2009). Activity of antibacterial compounds immobilised on montmorillonite. Appl Clay Sci.

[CR25] Mitik-Dineva N, Wang J, Truong VK, Stoddart P, Malherbe F, Crawford RJ, Ivanova EP (2009). *Escherichia coli, Pseudomonas aeruginosa*, and *Staphylococcus aureus* attachment patterns on glass surfaces with nanoscale roughness. Curr Microbiol.

[CR26] Neu HC (1992). The crisis in antibiotic resistance. Science.

[CR27] Ng C, Teoh S, Chung T, Hutmacher D (2000). Simultaneous biaxial drawing of poly (ϵ-caprolactone) films. Polymer.

[CR28] Nigmatullin R, Gao F, Konovalova V (2009). Permanent, non-leaching antimicrobial polyamide nanocomposites based on organoclays modified with a cationic polymer. Macromol Mater Eng.

[CR29] Parolo ME, Fernández LG, Zajonkovsky I, Sánchez MP, Bastion M (2011) Antibacterial activity of materials synthesized from clay minerals. In: Science against microbial pathogens: communicating current research and technological advances. Microbiology series, vol 3. Formatex, pp 144–151

[CR30] Pérez CJ, Alvarez VA, Mondragón I, Vázquez A (2008). Water uptake behavior of layered silicate/starch–polycaprolactone blend nanocomposites. Polym Int.

[CR37] Pranamuda H, Tokiwa Y, Tanaka H (1996). Physical properties and biodegradability of blends containing
poly (ε-caprolactone) and tropical starches. J Environ Polym Degrad.

[CR31] Qin Y, Zhuang Y, Wu Y, Li L (2016). Quality evaluation of hot peppers stored in biodegradable poly (lactic acid)-based active packaging. Sci Hortic.

[CR32] Rhim J-W, Hong S-I, Ha C-S (2009). Tensile, water vapor barrier and antimicrobial properties of PLA/nanoclay composite films LWT-Food. Sci Technol.

[CR33] Sauvet G, Dupond S, Kazmierski K, Chojnowski J (2000). Biocidal polymers active by contact. V. Synthesis of polysiloxanes with biocidal activity. J Appl Polym Sci.

[CR34] Senuma M, Tashiro T, Iwakura M, Kaeriyama K, Shimura Y (1989). Synthesis and antibacterial activity of copolymers having a quaternary ammonium salt side group. J Appl Polym Sci.

[CR35] Speranza G (2004). Role of chemical interactions in bacterial adhesion to polymer surfaces. Biomaterials.

[CR36] Stewart PS, Costerton JW (2001). Antibiotic resistance of bacteria in biofilms. Lancet.

[CR38] Venugopal J, Ramakrishna S (2005). Applications of polymer nanofibers in biomedicine and biotechnology. Appl Biochem Biotechnol.

[CR39] Yahiaoui F, Benhacine F, Ferfera-Harrar H, Habi A, Hadj-Hamou AS, Grohens Y (2015). Development of antimicrobial PCL/nanoclay nanocomposite films with enhanced mechanical and water vapor barrier properties for packaging applications. Polym Bull.

